# *In utero* Exposure to Maternal Chronic Inflammation Transfers a Pro-Inflammatory Profile to Generation F2 via Sex-Specific Mechanisms

**DOI:** 10.3389/fimmu.2020.00048

**Published:** 2020-02-13

**Authors:** Rozanne Charlene McChary Adams, Carine Smith

**Affiliations:** Department of Physiological Sciences, Stellenbosch University, Stellenbosch, South Africa

**Keywords:** infection, cytokine, glucocorticoid receptor, gender, transgenerational inheritance, periconception systemic inflammation

## Abstract

Generational transfer of maladaptations in offspring have been reported to persist for multiple generations in conditions of chronic inflammation, metabolic and psychological stress. Thus, the current study aimed to expand our understanding of the nature, potential sex specificity, and transgenerational plasticity of inflammatory maladaptations resulting from maternal chronic inflammation. Briefly, F1 and F2 generations of offspring from C57/BL/6 dams exposed to a modified maternal periconception systemic inflammation (MSPI) protocol were profiled in terms of leukocyte and splenocyte counts and cytokine responses, as well as glucocorticoid sensitivity. Overall, F1 male and female LPS groups presented with glucocorticoid hypersensitivity (with elevated corticosterone and increased leukocyte glucocorticoid receptor levels) along with a pro-inflammatory phenotype, which carried over to the F2 generation. The transfer of inflammatory and glucocorticoid responsiveness from F1 to F2 is evident, with heritability of this phenotype in F2. The findings suggest that maternal (F0) perinatal chronic inflammation resulted in glucocorticoid dysregulation and a resultant pro-inflammatory phenotype, which is transferred in the maternal lineage but seems to affect male offspring to a greater extent. Of further interest, upregulation of IL-1β cytokine responses is reported in female offspring only. The cumulative maladaptation reported in F2 offspring when both F1 parents were affected by maternal LPS exposure is suggestive of immune senescence. Given the potential impact of current results and the lack of sex-specific investigations, more research in this context is urgently required.

## Introduction

It is proposed by a large body of literature that adversities (i.e., infectious, metabolic, or psychological stress) during gestational development have a major impact on the long-term health outcomes of offspring into adulthood. Extensive research on human and animal studies showed that, in the context of maternal stress or physiological disturbances, adversity during gestation can result in long-term pathological outcomes in offspring. For example, in models such as poor nutrition and chronic social stress, in humans, altered serum concentrations of fatty acid binding protein 4 and adiponectin (1) were reported. In rodent models, altered corticosterone (CORT), prolactin, and oxytocin (2), intercellular adhesion molecule-1 (ICAM-1), granulocyte–macrophage colony-stimulating factor (GM-CSF), IL-18, progesterone, and vascular endothelial growth factor (VEGF) (3) were illustrated. Furthermore, these outcomes were demonstrated to exceed the first generation and to persist up to three generations downstream (4, 5). Moreover, the immune response in offspring (F1 to F3) is also altered by maternal exposure to psychological stress (6, 7), soluble glucocorticoid challenge (5, 8), or immune challenge (9–12) during gestation.

Although some of these studies investigated transgenerational inheritance of the altered phenotype by investigations up to F3, little information was gathered on the sex specificity of the changes maintained up to F3. Nevertheless, the fact that one group repeated reported a paternally transferred increase in glucocorticoid sensitivity in response to maternal gestational exposure to exogenous glucocorticoids (5, 8, 13), suggests that sex specificity indeed does exist. However, although many of the adaptive/maladaptive changes reported in offspring relate back to inflammation, a known etiological factor in most modern chronic disease states, very little data exist on the role of inflammation in this context.

From the limited data available, decreased capacity to mount an immune response to acute experimental infection was reported in F1 offspring to mothers acutely challenged with LPS during periconception (12). More in line with our context of low-grade inflammation in chronic disease etiology, chronic low-dose maternal LPS exposure during pregnancy and lactation resulted in a male-specific upregulation of blood leukocyte count and hypothalamic inflammatory cytokines in F1 offspring, in the absence of further specific stimulus (i.e., basally) (10). F1 offspring is not indicative of generational inheritance because of the potential confounding influence of the *in utero* microenvironment (e.g., LPS-associated damage to the placenta) (14, 15). By employing a model capable of reflecting maternal generational transfer, we recently reported in mice that experimentally induced chronic maternal gestational inflammation resulted in altered inflammatory profiles in at least two generations of offspring (16), which is consistent with an interpretation of multigenerational inheritance. Importantly, in terms of preventative treatment, in line with the “fetal origins of adult disease” hypothesis by Barker (17), recent publications described successful *in utero* treatment of inherited diseases, such as brittle bone disease (18), hemophilia (19), and Gaucher's disease (20). Thus, given the phenomenon of generational transfer of maladapted physiology already demonstrated in the context of stress and obesity, both conditions with an inflammatory component, the option for corrective intervention *in utero* may be more widely applicable than just congenital disorders. Indeed, the possibility of decreasing risk for the development of especially non-communicable diseases—which are currently the number one cause of mortality world-wide (21)—by addressing fetal inflammatory phenotype, will have huge impact on the health sector globally. However, more detailed information on the nature and generational plasticity of specific mechanisms by which maladaptive inflammatory responses may occur is required.

The current gap, in terms of the sex dependence on these inherited inflammatory maladaptations, is a major obstacle in the development of strategies such as the *in utero* corrective intervention just mentioned and also in terms of preventative medicine practices. Our previously published study reflected the transgenerational adaptations that occur in chronic gestational LPS administration and its effect on two subsequent generations of offspring (16). In an attempt to bridge this gap in literature, we here expand on our previous study in two ways to determine potential sex differences in the pro-inflammatory maladaptation previously reported and potentially delineate sex-specific heritability. First, we present a reanalysis of a portion of the data previously published (16) to reflect sex specificity of maladaptive inheritance. Second, we report novel data of cumulative inheritance in F2 offspring born from two LPS-affected (F1) parents.

## Materials and Methods

### Experimental Animals

Ethical clearance for the study was obtained from the Stellenbosch University Animal Research Ethics Committee (Ref # SU-ACUM14-00004). The C57/BL/6 mouse strain was used for the current study. Mouse siblings were housed in groups of five, separated by sex, with the exception of late stage pregnant and lactating mothers who were individually housed. All animals were subjected to temperature-controlled and humidity-controlled conditions (22°C and 40% humidity) with a 12-h dark–light cycle, for the duration of the study, with *ad libitum* access to standard rodent chow and water, as well as cage enrichment in the form of nesting material and tubing.

**Figure 1:**
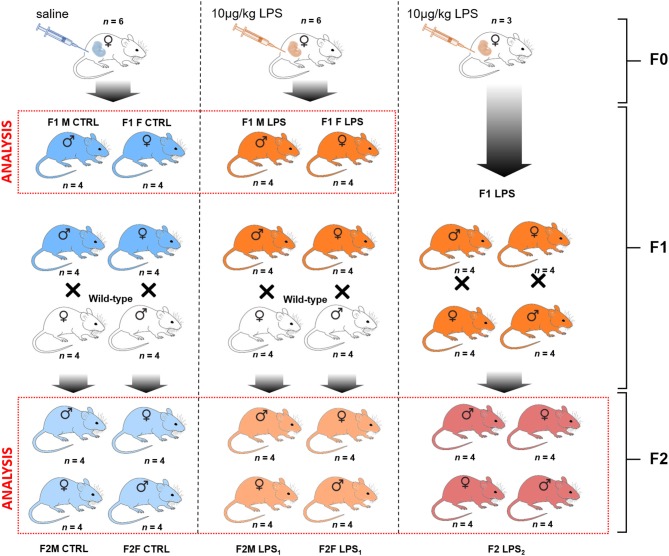
Breeding paradigm for control and LPS-affected mice. The *n* represents the number of mice for each subsequent intervention group and sex per F1 and F2 generations used for analysis (demarcated in the red boxes).

The breeding protocol followed is visually presented in Figure 1. For the initial breeding of the F0 generation, 7-week-old dams were naturally crossed with age-matched studs. The following day, plug-positive dams were randomized to receive either LPS [from *E. coli* (Sigma-Aldrich, USA)] at 10 μg/kg body mass by intraperitoneal injection (LPS group), prepared in 0.9% saline, or 0.9% saline only (CTRL group), at a final volume of 50 μl. The F0 mothers were administered their respective injections every 7 days until the end of the gestation period (on average 20 days) and after this time point, no further interventions were given to either mothers or resulting F1 and F2 offspring for the duration of the study. As a safety precaution, the F0 dams were monitored for adverse reactions post-administration of the intervention.

The first generation of offspring, F1, was bred to 7 weeks of age and were then either terminated via cervical dislocation for sample collection or naturally crossed with an age-matched wild-type (i.e., unaffected) C57/BL/6 mouse to produce the second generation of offspring, F2. In other words, an F1 LPS-affected or F1 CTRL female was crossed with a wild-type male C57/BL/6 mouse that had received no form of intervention. Similarly, an F1 LPS affected or F1 CTRL male was crossed with a wild-type female C57/BL/6 mouse that had received no form of intervention. Additionally, a group of F1 LPS-affected males were crossed with (non-sibling) F1 LPS-affected females to produce an F2 generation with both parents affected by LPS. Grouping of animals for either termination or further breeding were performed randomly by an independent person. Information on litter size is presented in Table 1. All F2 offspring were again bred to 7 weeks of age and then terminated by cervical dislocation, for sample collection and analysis. Thus, for both F1 and F2, all animals were terminated at the same age.

**Table 1:** Gestation data for LPS-exposed and LPS-affected mice.

	**No. of animals**	**Mating partner**	**Gestation length (days)**	**Total no. of offspring**	**Mean litter size**	**Male: female ratio**
F0 F CTRL	5	-	20	33	6.6 ± 0.98	1.3 ± 0.35
F0 F LPS	5	-	20	30	6.0 ± 0.94	1.5 ± 0.47
F1 M CTRL	4	WT female	20	19	4.8 ± 0.47	1.4 ± 0.53
F1 M LPS	4	WT female	20	22	5.5 ± 0.50	1.0 ± 0.38
F1 F CTRL	4	WT male	20	22	5.5 ± 0.23	1.3 ± 0.29
F1 F LPS	4	WT male	20	24	6.0 ± 1.30	1.5 ± 0.55
F1 F LPS	3	F1 LPS male	20	20	10.0 ± 1.10^a^	1.3 ± 0.03

*Values are depicted as mean ± SEM*.

a *Significantly different from all other groups (at least p < 0.05)*.

For analysis, the designated groups were then separated into male and female mice. In the F1 generation, mice were grouped as F1 Female (F1F) and F1 Male (F1M) group, for the CTRL and LPS intervention groups. In the F2 generation, offspring were classified as follows for the CTRL groups: F2M Male CTRL—male offspring resulting from F1 Male CTRLx wild-type C57/BL/6 female; F2M Female CTRL—female offspring resulting from F1 Male CTRLx wild-type C57/BL/6 female; F2F Male CTRL—male offspring resulting from F1 Female CTRL x wild-type C57/BL/6 male; F2F Female CTRL—female offspring resulting from F1 Female CTRL x wild-type C57/BL/6 male.

The classification for the F2 LPS group is as follows: F2M Male LPS_1_–male offspring resulting from F1 Male LPSx wild-type C57/BL/6 female; F2M Female LPS_1_–female offspring resulting from F1 Male LPSx wild-type C57/BL/6 female; F2F Male LPS_1_–male offspring resulting from F1 Female LPSx wild-type C57/BL/6 male; F2F Female LPS_1_–female offspring resulting from F1 Female LPSx wild-type C57/BL/6 male.

The classification for the F2 LPS offspring from F1 LPS male and female parents is as follows: F2 Male LPS_2_–male offspring resulting from F1 Male LPS x F1 Female LPS; F2 Female LPS_2_–female offspring resulting from F1 Male LPS group x F1 Female LPS.

### Sample Collection

Whole blood was collected via cardiac puncture and transferred into K_2_EDTA microtubes for full and differential blood counts on the Cell-Dyne 3700CS hemocytometer (Abbott Diagnostics, USA). Plasma was collected from the whole blood samples and analyzed for basal corticosterone (CORT) concentrations by quantitative ELISA (Demeditec Corticosterone rat/mouse ELISA, Demeditec Diagnostics, Germany). Concentrations were calculated on a six-point standard curve with a logistic regression algorithm. The assay detection range was 6.1–2,250 ng/ml.

Spleens were collected in ice-cold complete RPMI 1640 (cRPMI) medium, consisting of 10% fetal bovine serum, 1% penicillin–streptomycin, and 1% gentamicin, for isolation of splenocytes.

### Cell Preparation

Spleens were mechanically dissociated and strained through a 70-μm cell strainer to acquire single-cell suspensions. Red blood cells were lysed for 5 min at room temperature using ammonium–chloride–potassium (ACK) lysis buffer and the splenocytes counted on the Countess Cell Counter (Thermofisher Scientific, USA). Splenocyte counts were then adjusted to 1.0 × 10^7^ cells per ml in cRPMI.

### *Ex vivo* Cytokine Stimulation

For *ex vivo* stimulation, splenocytes were cultured at a concentration of 1 × 10^6^cells/ml either in the absence or presence of LPS (1 μg/ml) for 18 h, at 37°C, 5% CO_2_. The supernatant was collected to determine cytokine profile. The MAP Mouse Cytokine/Chemokine Magnetic Bead panel (Millipore, USA) was used to assess the levels of IL-1β, IL-6, IL-10, TNF-α, and IFN-γ. The samples were prepared in duplicate, as per manufacturer's instructions, and run on the Bioplex 200 system (Biorad, USA) equipped with Bio-Plex Manager™ software. Cytokine concentrations were automatically calculated using a six-point standard curve fitted with a five-parameter logistic regression algorithm. The kit's lowest detection thresholds were as follows: IL-1β, 5.4 pg/ml; IL-6, 1.1 pg/ml; TNF-α, 2.3 pg/ml, and IL-10, 2.0 pg/ml.

### Immunocytochemistry

Glucocorticoid receptor expression was assessed in splenic leukocyte subsets by flow cytometry. After isolation, the splenocytes were stained with Zombie Aqua Fixable Viability Dye (Biolegend) for 30 min at room temperature and washed with 1× DPBS (Gibco, USA). Mouse FC Block (BD Biosciences) was then added for 5 min before cell surface proteins were labeled with an antibody cocktail containing the following antibodies: Brilliant Violent 421 (BV421)-conjugated anti-NK1.1 (Biolegend); Fluorescein isothiocyanate (FITC)-conjugated anti-TCRβ (BD Biosciences); PE–CF594-conjugated anti-F4/80 (BD Biosciences); Peridinin chlorophyll (PerCP)–Cy5.5-conjugated anti-CD11b (BD Biosciences); and allophycocyanin–Cy7 (APC–Cy7)-conjugated anti-Ly6G (BD Biosciences). After 30 min of incubation, cells were washed twice with staining buffer [1X DPBS containing 5% bovine serum albumin (Invitrogen, USA) and 1% sodium azide (Sigma Aldrich, USA)]. The cells were fixed and permeabilized using the Cytofix/Cytopermkit (BD Biosciences, USA) as per manufacturer's instruction. For intracytoplasmic staining of the GR receptor, cells were incubated with the anti-NR3C1 antibody [Alexafluor 647-conjugated anti-NR3C1 (Novus Biologicals)] for 30 min. After incubation, cells were washed twice and resuspended in staining buffer before acquisition on the flow cytometer.

### Flow Cytometry

The flow cytometric analysis was performed at the Central Analytical Facilities' Fluorescence Imaging Unit at Stellenbosch University. The gating strategy employed is illustrated in Supplemental Figure 1. All prepared samples were analyzed on the BD FACSAriaIIu flow cytometer (BD Biosciences, USA) with BD FACSDiva™ ver. 8.01 software for data acquisition and analysis.

For the leukocyte glucocorticoid receptor expression analysis, a minimum of 200,000 and a maximum of 500,000 live, gated, singlet events were acquired from each sample tube. Relative glucocorticoid receptor (NR3C1) expression was assessed in T-lymphocytes (TCRβ+ NK1.1–), NKT lymphocytes (TCRβ+ NK1.1+) NK cells (TCRβ- CD11b+ NK1.1+), neutrophils (TCRβ- CD11b+ Ly6G+), monocytes (TCRβ- CD11b+ F4/80–), and macrophages (TCRβ- CD11b+ F4/80+), as identified by their specific marker expression. The gating strategy was previously described in more detail (16).

For each respective assay, samples were run using application settings to verify MFI targets, and compensation was performed with every run. All data files were further analyzed in FlowJo™ v10.4.1 and reported as percentage positive (frequency) and median fluorescent intensity (MFI).

### Statistical Analysis

Data are reported as means and standard errors of the mean. Data were analyzed using the Statistica software 13.4 (Statsoft, USA), and graphs were generated in a GraphPad Prism 7 (GraphPad Software Incorporated, USA). The F1 and F2 datasets were analyzed separately using a two-way ANOVA to assess the main effects of LPS exposure *in utero* (F1) or ancestral LPS exposure (F2) between sexes for each generation. To avoid interpretation of seasonal immune differences between F1 and F2 generations (22, 23) as resulting from prior LPS exposure or generational differences, the two generations were not directly compared with each other. A Fisher's *post-hoc* analysis was performed to compare individual group means for statistical differences. All data are presented as mean ± SEM, and *p* < 0.05 was regarded as significant.

## Results

### Body Mass Changes in Responses to Generational LPS Exposure

In terms of body mass, in F1, a sex-specific difference was seen, with males generally heavier than females, which was not unexpected. However, no effect of *in utero* LPS was evident (Table 2).

**Table 2:** Bodymass (g) for the F1 generation for LPS-affected and CTRL groups.

	**F1 males**	**F1 females**
**CTRL**	29.6 ± 0.86	23.7 ± 1.93^a^
**LPS**	28.5 ± 0.34	21.7 ± 0.65^b^

*Data are depicted as mean ± SEM (n = 4–6 per group)*.

a *Significantly different from F1 Male CTRL (p < 0.05)*.

b *Significantly different from F1 Male LPS (p < 0.05)*.

In the F2 generation, males still maintained a higher body mass than females (Table 3). In addition, F2 females generally maintained a similar body mass irrespective of parental LPS exposure. In contrast, F2 male offspring from LPS-affected mothers exhibited a higher body mass when compared to their controls. In addition, F2 males from two LPS-affected parents had even higher body mass.

**Table 3:** Body mass (g) of generation F2.

	**F2M males**	**F2M females**	**F2F males**	**F2F females**	**F2 LPS_**2**_ males**	**F2 LPS_**2**_ females**
CTRL	22.9 ± 0.70	21.0 ± 0.41	20.80 ± 0.89	19.3 ± 0.28	-	-
LPS	24.4 ± 1.69	22.2 ± 1.91	26.4 ± 1.90^a^	21.4 ± 0.33	28.5 ± 0.34^b^	21.9 ± 0.23^c^

*Data are depicted as mean ± SEM (n = 4 per group)*.

a *Body mass significantly higher than F2F Male CTRL (p = 0.05)*.

b *Body mass significantly higher than all other male groups (p < 0.05 at least)*.

c *Body mass significantly lower than F2 LPS_2_ Males (p < 0.05)*.

### Changes in Leukocyte Distribution in Circulation vs. Spleen

#### Peripheral Leukocytes

**Figure 2:**
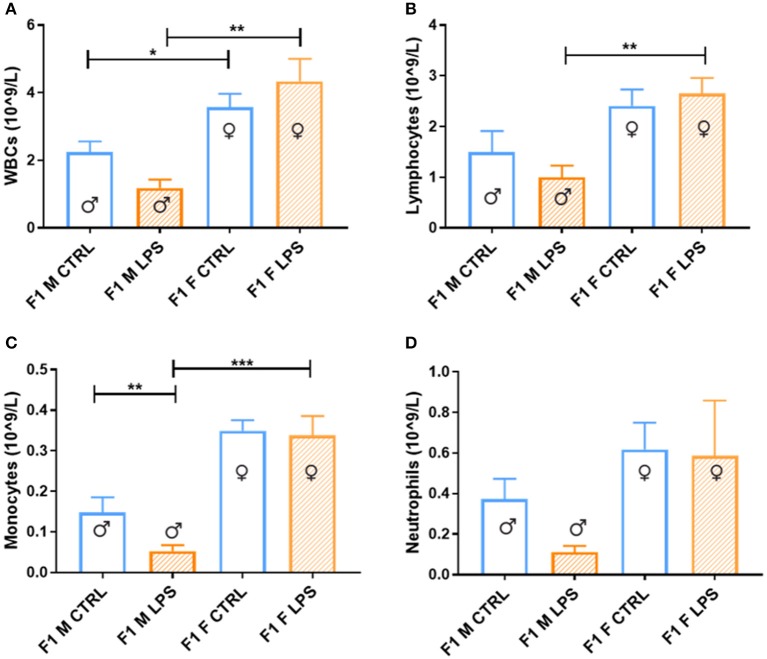
Peripheral blood leukocytes between the saline Control and LPS-affected groups for male and female F1 groups. The data is depicted as the mean ± SEM. The cell subset comparisons for F1M and F1FControl and LPS affected are depicted as follows: **(A)** total WBCs, **(B)** lymphocytes, **(C)** monocytes, and **(D)** neutrophils (*n* = 4 per group). *p < 0.05; **p < 0.01; ***p < 0.001.

Generally, in generation F1, females displayed a higher total peripheral blood leukocyte count than males (Figure 2), independent of maternal chronic inflammation. In addition, in the LPS-affected group, F1 males displayed further significant decreases in leukocyte counts in comparison to their female counterparts.

When looking closer at specific leukocyte subsets, a similar trend emerges more noticeably with the lymphocyte, monocyte, and neutrophil counts, where a male-specific reduction in peripheral cell count is associated with exposure, contributing to relatively higher peripheral leukocyte counts in LPS-affected females.

**Figure 3:**
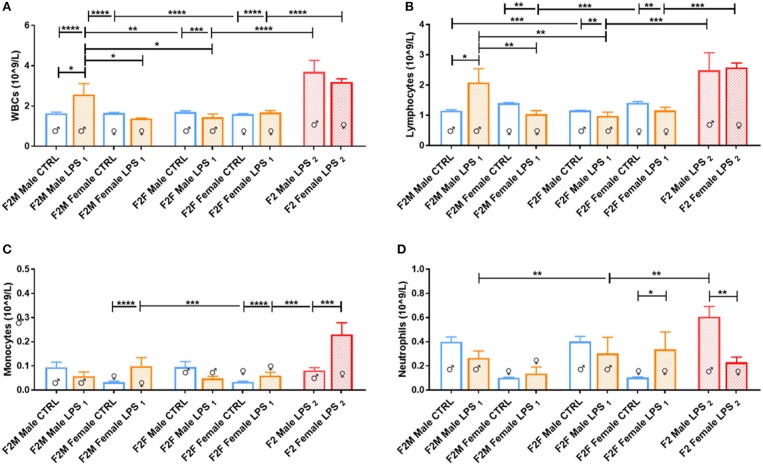
Peripheral blood leukocytes for male and female F2 Control, LPS_1_ and LPS_2_ groups. The data is depicted as mean ± SEM. The cell subset comparisons are depicted as follows: **(A)** total WBCs, **(B)** lymphocytes, **(C)** monocytes, and **(D)** neutrophils (*n* = 4 per group). Significance is as follows: *p < 0.05; **p < 0.01; ***p < 0.001; ****p < 0.0001.

In the F2 generation, differences were again observed between the sexes (Figure 3). The F2M LPS males displayed significantly higher total WBCs in peripheral blood in comparison to the F2M controls, as well as in comparison to the F2M Female LPS and F2F Male LPS group. This effect may be because of specifically higher lymphocyte and neutrophil counts, which are the two subpopulations that primarily showed this pattern as well. Interestingly, F2F Male LPS group also showed significantly higher neutrophil counts in comparison to their F2F Male controls, while females did not—this is in line with an interpretation of sex specificity in the response to LPS exposure.

When comparing the F2 LPS_2_ male and female groups, both groups had substantially higher total WBCs in both sexes compared to both the offspring from a single affected parent (LPS_1_) and those from unaffected parents (CTRL). In addition, in this group, while both sexes exhibited higher lymphocyte numbers, the F2 LPS_2_ males also displayed a sex-specific increase in monocyte and neutrophil counts, while the F2 LPS_2_ females had significantly higher monocyte counts in the peripheral blood.

#### Splenic Leukocytes

Comparable to leukocyte counts in peripheral blood, females exhibited much higher splenic leukocyte counts than males, for the majority of leukocyte subpopulations assessed (Table 4). Maternal LPS exposure resulted in increased total leukocyte counts for both sexes when compared to their CTRL groups, but only reached statistical significance in the F1 males. In both sexes, the maternal LPS exposure also drastically increased lymphocyte counts, with males showing the largest incremental response. This suggests that the change in total leukocyte count is mainly due to the increase in lymphocyte numbers in the spleen.

**Table 4:** Differential leukocyte counts in spleen between the different sexes for the F1 generation for LPS-affected and CTRL groups.

	**WBCs (10^9/L^)**	**Lymphocytes (10^9^/L)**	**Monocytes (10^9^/L)**	**Neutrophils (10^9^/L)**
F1 M CTRL	1.20 ± 0.07	0.43 ± 0.03	0.25 ± 0.02	0.30 ± 0.06
F1 M LPS	4.57 ± 0.42^a^	3.59 ± 0.23^a^	0.43 ± 0.12	0.15 ± 0.04
F1 F CTRL	3.65 ± 0.88^a^^,^^c^	2.63 ± 0.70^a^^,^^c^	0.35 ± 0.10	0.27 ± 0.12
F1 F LPS	4.82 ± 0.44	4.07 ± 0.35^b^	0.36 ± 0.04	0.12 ± 0.01

*Data are depicted as mean ± SEM (n = 4 per group). For each group, cell count is depicted at 10^9^/L*.

a *Significantly different from F1 M Control (at least p ≤ 0.05)*.

b *Significantly different from F1 F Control (p < 0.001)*.

c *Significantly different from F1 M Control (p < 0.01)*.

In the F2 generation, LPS-affected animals again had significantly affected splenic leukocyte counts (Table 5). In the F2 offspring, leukocyte counts for the majority of subsets in LPS-affected groups were consistently reduced, with no specific sex specificity. Only one exception was observed: the total leukocyte count in the LPS-affected F2F males did not show a decrease—this can potentially be ascribed to somewhat higher lymphocyte and significantly higher neutrophil counts in this group only. The significance of this remains to be validated.

**Table 5:** Differential leukocyte counts in spleen between the different sexes for the F2 generation for the Control, LPS_1_, and LPS_2_ groups.

			**WBCs (10^9^/L)**	**Lymphocytes (10^9^/L)**	**Monocytes (10^9^/L)**	**Neutrophils (10^9^/L)**
Offspring To F1 males	F2M males	F2M male CNTRL	2.28 ± 0.33	1.43 ± 0.06	0.19 ± 0.01	0.3 ± 0.04
		F2M Male LPS_1_	1.76 ± 0.02^f^	1.32 ± 0.08^f^	0.15 ± 0.03^f^	0.07 ± 0.007^a^
	F2M females	F2M female CNTRL	3.32 ± 0.22^a^^,^^g^	2.41 ± 0.14^a^	0.27 ± 0.05	0.3 ± 0.05^g^
		F2M female LPS_1_	1.24 ± 0.08^b^	0.81 ± 0.10^b^	0.14 ± 0.006^b^	0.08 ± 0.004^b^^,^^g^
Offspring to F1 females	F2F males	F2F male CNTRL	2.00 ± 0.11	1.05 ± 0.06	0.27 ± 0.07	0.26 ± 0.02
		F2F male LPS_1_	2.30 ± 0.67	1.78 ± 0.74	0.12 ± 0.01^c^^,^^f^	0.11 ± 0.03^c^
	F2F females	F2F female CNTRL	2.38 ± 0.06	1.42 ± 0.07	0.26 ± 0.03	0.2 ± 0.01^2,7^
		F2F female LPS_1_	1.19 ± 0.16^d^^,^^e^^,^^f^	0.88 ± 0.23	0.13 ± 0.07^d^	0.05 ± 0.01^d^^,^^f^
Offspring of F1 LPS male x F1 LPS female	F2 LPS_2_	F2 male LPS_2_	2.38 ± 0.17	1.70 ± 0.167	0.26 ± 0.03	0.17 ± 0.02
		F2 female LPS_2_	2.14 ± 0.09	1.13 ± 0.08^f^	0.18 ± 0.03	0.85 ± 0.28

*The affected parental lineage is described as offspring of F1 Males or F1 Females, which describes whether the affected (LPS-affected or saline CNTRL) parental group is male or female, respectively. The groups are further grouped into male and female for their respective parental group. Data are depicted as mean ± SEM (n = 4 per group). For each group, cell count is depicted at 10^9^ cells/L*.

a *Significantly different from F2M Male CTRL (at least p ≤ 0.05)*.

b *Significantly different from F2M Female CTRL (at least p ≤ 0.05)*.

c *Significantly different from F2F Male Control (at least p ≤ 0.05)*.

d *Significantly different from F2F Female Control (at least p ≤ 0.05)*.

e *Significantly different from F2F Male LPS_1_ (at least p ≤ 0.05)*.

f *Significantly different from F2 Male LPS_2_ (at least p ≤ 0.05)*.

g *Significantly different from F2 Female LPS_2_ (at least p ≤ 0.05)*.

F2 LPS_2_ splenic total WBCs did not show much response to LPS after exposure in both parental lines. F2 LPS_2_ males showed somewhat higher WBC counts than F2M and F2F LPS and CTRL males, as well as F2 LPS_2_ females. This was attributed to the significantly increased lymphocyte count seen in this group.

### Adaptations in Plasma Corticosterone Concentrations

**Figure 4:**
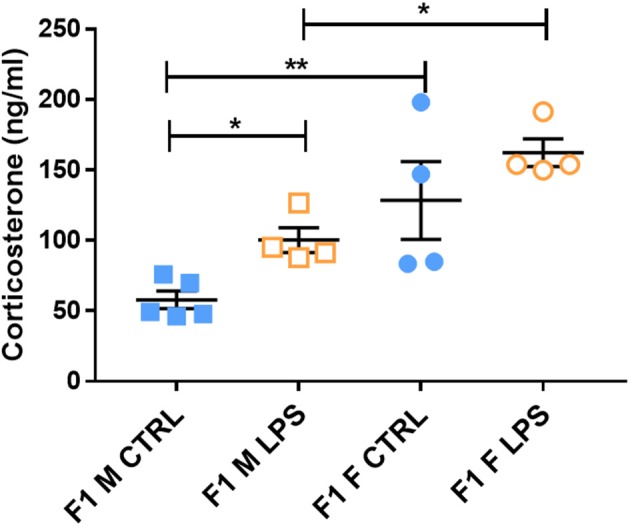
Basal plasma corticosterone concentrations in LPS-affected and saline controls in the F1 generation. All data are depicted as mean ± SEM (*n* = 4 per group). Significance is as follows: *p < 0.05; **p < 0.01.

The majority of F1 females showed higher CORT levels in plasma when compared to males, regardless of LPS exposure, pointing toward a physiological difference between sexes (Figure 4). Furthermore, LPS-affected F1 males exhibited significantly higher CORT levels when compared to male controls, while the F1 females did not exhibit a CORT response to maternal periconception chronic inflammation.

**Figure 5:**
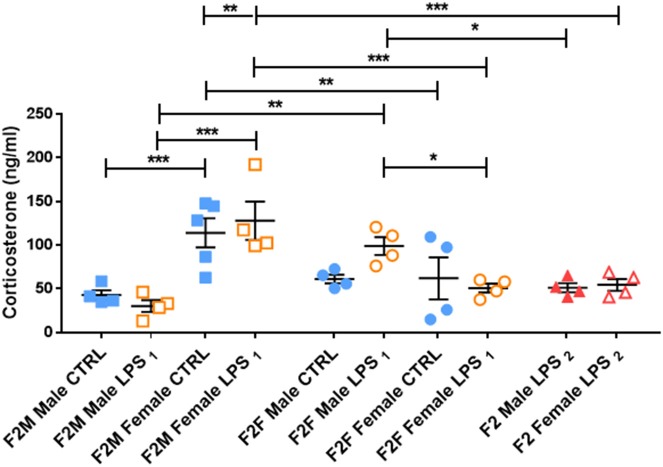
Basal plasma corticosterone concentrations in the Control, LPS_1_ and LPS_2_ groups in the F2 generation. All data are depicted as mean ± SEM (*n* = 4 per group). Significance is as follows: *p < 0.05; **p < 0.01, ***p < 0.001.

This male-specific effect was perpetuated to the offspring of F1 Males, where basal plasma CORT concentrations in F2M females were again higher than that of F2M males, regardless of LPS (Figure 5). However, unlike in F1 males, F2M LPS-affected males did not exhibit upregulated CORT levels. Of interest, the F2F Male LPS group seemed to have somewhat higher CORT levels than their controls (although not statistically significant), resulting in statistically significantly higher CORT when compared to F2F Female LPS (Figure 5). In addition, while the F2F Male LPS group had higher plasma CORT levels when compared to the F2M Male LPS group, CORT levels in F2F Female LPS were significantly lower than that of F2M Female LPS.

F2 LPS_2_ males and females seemed to have blunted CORT levels, as their values were comparable to their respective F2F and F2M CTRL groups. Furthermore, F2 LPS_2_ male CORT levels were significantly lower than that of the F2F LPS males. F2 LPS_2_ females had CORT levels significantly lower than the F2F and F2M LPS females as well as the F2M CTRL females.

### Basal Glucocorticoid Receptor Expression in Splenic Leukocytes

**Figure 6:**
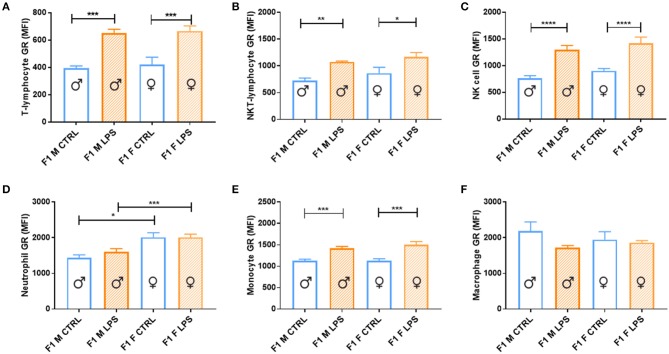
Splenocyte leukocyte subset expression of glucocorticoid receptor for male and female CTRL and LPS groups within the F1 generation. The glucocorticoid receptor (GR) expression comparisons are depicted as follows: **(A)** T lymphocytes, **(B)** NKT lymphocytes, **(C)** NK cells, **(D)** neutrophils, **(E)** monocytes, and **(F)** macrophages (*n* = 4 per group). All data are depicted as mean ± SEM (*n* = 4 per group). Significance is as follows: *p < 0.05; **p < 0.01; ***p < 0.001; ****p < 0.0001.

Both sexes in the F1 generation exhibited significant upregulation of GR expression in response to LPS for most of the cell subtypes analyzed [T lymphocytes, innate-like lymphocytes (NKT lymphocytes and NK cells) and monocytes (Figure 6)]. In contrast, both macrophage and neutrophil GR seemed unaffected by LPS, with neutrophils being the only cell subtype to display a sex-specific effect, with a higher GR expression in F1 females, independent of LPS exposure.

**Figure 7:**
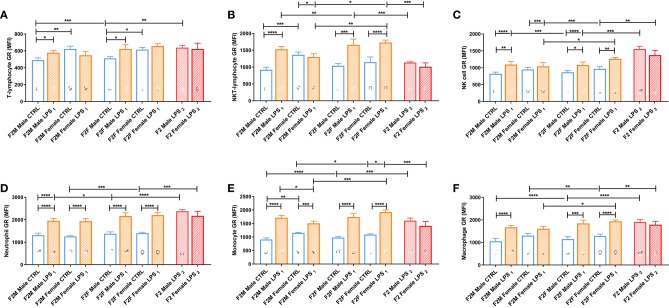
Splenocyte leukocyte subset expression of the glucocorticoid receptor for male and female CTRL, LPS_1_, and LPS_2_ groups within the F2 generation. The glucocorticoid receptor expression comparisons are depicted for the following leukocyte subsets: **(A)** T lymphocytes, **(B)** NKT lymphocytes, **(C)** NK cells, **(D)** neutrophils, **(E)** monocytes, and **(F)** macrophages (*n* = 4 per group). All data are depicted as mean ± SEM. Significance is as follows: *p < 0.05; **p < 0.01; ***p < 0.001; ****p < 0.0001.

GR expression levels in F2 offspring differed with parental LPS exposure and were dependent on the affected lineage (F1 male or female) (Figure 7). In the F2M group, only males from the LPS-affected fathers had significantly higher GR expression in T lymphocytes, NKT lymphocytes, NK cells, monocytes, and macrophages, compared to the F2M CTRL, while in females from the LPS-affected fathers, GR expression was only upregulated in neutrophils. In the F2F group, offspring from the affected F1 females were both sexes who seemed to increase GR expression in most of the cell subtypes, with LPS-associated changes in GR expression levels seeming to mirror that illustrated for F1. This indicates that GR adaptation was maintained into F2. When comparing F2F vs. F2M, the GR expression levels for all leukocyte subsets, except neutrophils, were generally significantly higher in the F2 LPS female group than in the F2 M LPS group.

Looking at the F2 LPS_2_ group, this group showed no specific sex differences between their male and female counterparts for all the parameters assessed. Furthermore, in the second generation (F2), both parents' exposure to LPS only affected GR expression for monocytes, NK cells, and NKT cells, when compared to single-parent exposure (male or female). Of significance, NKT-lymphocytes and monocytes downregulated GR expression, and NK cell GR expression was upregulated with dual LPS-affected in both F2 LPS_2_ males and females, which was especially lower in the F2 LPS_1_ females.

### Splenocyte Cytokine Response

**Figure 8:**
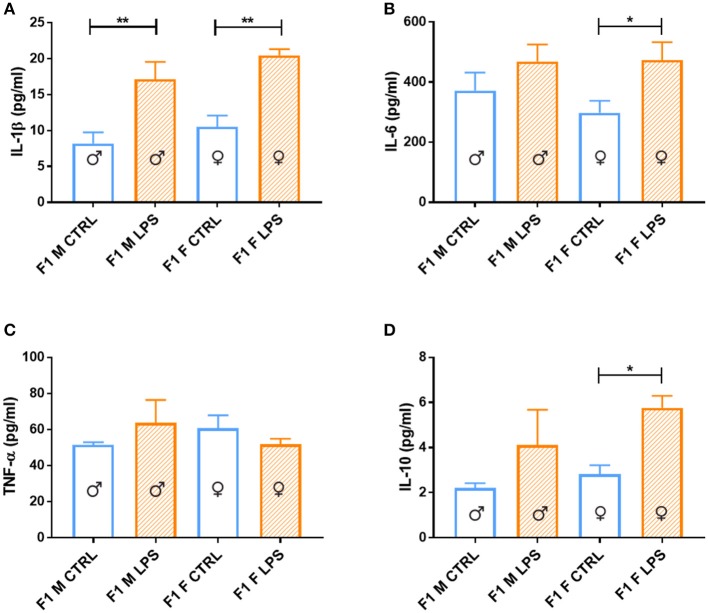
Splenocyte LPS-induced *ex vivo* cytokine responses between the male and female CTRL and LPS groups for F1 generation. Concentrations of **(A)** IL-1β, **(B)** IL-6, **(C)** TNF-α, and **(D)** IL-10 were analyzed after 18 h of incubation with 1 μg/ml of LPS. Significance is as follows: *p < 0.05; **p < 0.01.

**Figure 9:**
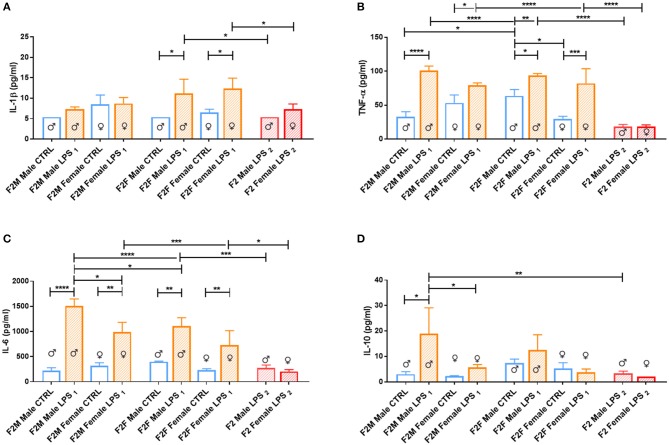
Splenocyte LPS-induced *ex vivo* cytokine responses between the male and female CTRL and LPS groups for F1 generation. Concentrations of **(A)** IL-1β, **(B)** IL-6, **(C)** TNF-α, and **(D)** IL-10 were analyzed after 18 h of incubation with 1 μg/ml of LPS. Significance is as follows: *p < 0.05; **p < 0.01; ***p < 0.001; ****p < 0.0001.

When considering the cytokine responses to acute *in vitro* LPS challenge, in F1 offspring (to primary affected mothers), acute LPS challenge elicited a significant IL-1β response (Figure 8A) in both sexes. This response was maintained in F2, but only for the offspring of F1 LPS-affected mothers (F2F LPS), while in F2M, this hyper-response was not evident (Figure 9A).

IL-6 showed a sex-specific increase in F1 LPS females compared to controls (Figure 8B), but TNF-α production was similar to F1 CTRL group for both sexes (Figure 8C). However, in F2, LPS-affected individuals all exhibited an exacerbated IL-6 and TNF-α response when compared to controls (Figures 9B,C).

In terms of anti-inflammatory cytokine response, F1 LPS-affected males seemed to mount relatively normal IL-10 responses to acute LPS challenge, while the females exhibited exacerbated responses to acute stimulation (Figure 8D). In the F2 group, the F2F LPS females had a normal IL-10 response. However, all other LPS-affected F2 offspring showed significantly increased IL-10 responses relative to controls, although this was only significant for the F2M male LPS group (Figure 9D).

Splenocytes from F2 LPS_2_ offspring seemed unable to elicit proper responses with LPS stimulation for all the cytokines assessed, when compared to the offspring of the single LPS-affected parents. TNF-α and IL-6 concentrations from F2 LPS_2_ males and females were significantly lower than the F2 LPS_1_ sex-specific counterparts. No difference was observed when considering other cytokines assessed.

## Discussion

The current study explored the generational transfer of HPA-axis- and immune adaptations across two generations of offspring, with the aim of delineating their possible sex-specific inheritance into subsequent generations. We expand on a previous report from our group (16) with the aim of delineating sex differences in the risk of disease vulnerability, as well as the potential of these effects to be cumulative. Our current *in vivo* mouse model of chronic maternal inflammation, described in Adams and Smith (16), is a modification of the maternal periconception systemic inflammation (MPSI) protocol (a single LPS injection at the start of gestation) established by Williams et al. (12)—our protocol entails weekly (every 7 days, starting immediately following plug-positive confirmation) LPS injections throughout the gestational period, to achieve consistent maternal exposure to the affecting agent during gestation. LPS administered intraperitoneally during gestation is known to not cross the placental barrier. However, the direct placental damage and subsequent fetal damage because of maternal LPS intervention was previously shown to persist into adulthood (15). Furthermore, the primordial germ cells present in the F1 generation, from which the F2 generation will arise, are affected by the F1 *in utero* exposure and result in maladaptive programming that may affect subsequent generations (24, 25). The extent of this plasticity remains to be determined in later generations. The current data corroborates the findings reported by Fricke et al. (14) and expands on it to include the F2 generation.

We report several sex-specific maladaptations resulting from gestational chronic maternal inflammation, some of which are maintained or even exacerbated in the second generation of offspring. We further add to the data by reporting on the cumulative maladaptations resulting from combining F1 LPS-affected male and female lineages, which severely impacted HPA-axis- and immunological functionality.

### Immune Adaptation to LPS Exposure in the F1 vs. F2 Generation

Generally, chronic stress and inflammation results in the characteristic upregulation of hematopoiesis and increased circulating leukocytes (10, 26, 27), which we similarly reported in the F0 female generation in our previous study (16). We showed that F0 dams exposed to LPS throughout gestation maintained raised leukocyte counts several weeks after the last administration of the low-dose (10 μg/kg) LPS, showing persistence in the inflammatory hematopoiesis. Here we demonstrate that this effect was modulated in the F1 offspring and exhibited sex dependence. Most notably, the leukocyte hypo-response was evident in F1 males and not females. In contrast, Dudele and colleagues reported a considerably higher WBC count in their model of chronic MSPI (10). This may be attributed, in part, to their model, which was designed to sustain LPS-induced low-grade inflammation throughout gestation and lactation, thus predisposing the offspring to longer exposure to inflammatory mediators. Interestingly, similar to our findings, repeated social stress also resulted in a male-specific reduction in peripheral blood leukocytes (28). This is in line with the glucocorticoid hypersensitivity reported in males in the literature (8) and reflected in our own data. In terms of cytokine responses, and similar to current results, Dudele et al. showed that their F1 male offspring displayed chronic immune activation (pro-inflammatory cytokine mRNA expression in liver tissue; increased hypothalamic inflammatory cytokines) that persisted at least up to 25 weeks of age (mature adulthood in mice). Of specific relevance in the context of generational transfer of inflammation, the demonstrated maladaptation to LPS exposure was similar to that seen in a peer group fed a high-fat diet in the same study (10). The current study further expands by also reporting a picture of immune activation in affected F1 males. In our model, circulating peripheral blood neutrophil counts were relatively depleted in the F1 LPS-affected offspring, suggesting their sequestration into the tissue. This interpretation was supported by relatively higher leukocyte counts in the spleen, compared to saline controls. This is in line with results reported in mouse models of chronic psychological stress (e.g., repeated social defeat) (29, 30), although the current study is the first to illustrate this in response to MSPI and offspring carryover.

We further expand on the current existing literature by identifying subsequent changes in the resulting F2 generation. Surprisingly, all four single-parent (LPS_1_) LPS-affected groups exhibited a general depletion of leukocytes from the spleen along with a raised leukocyte profile in the peripheral circulation, suggesting their trafficking from spleen to blood and possibly into tissue, an effect that seemed sex independent. This result, given the sex-dependent maladaptation evident in F1 males, cannot be explained from immune data alone. Rather, other factors, such as known changes in endogenous anti-inflammatory feedback capacity, may come into play.

In terms of potential for cumulative maladaptive changes, the F2 LPS_2_ offspring exhibited sex-dependent compartmental redistribution of cells, with male neutrophils depleting in the periphery in favor of increased sequestration in the spleen, while in contrast, female monocytes seemed to remain in large numbers in the periphery to result in relative depletion in the spleen.

In terms of functional immune capacity, both LPS_1_-affected sexes in F1 exhibited increased IL-1β responses to LPS stimulation in *ex vivo* culture, which is in line with available literature available on circulating plasma levels (14, 15) as well as data reported for a similar *ex vivo* stimulation model in F1 primates born to TLR3 agonist exposed mothers (31). In addition, when considering F2, the increased IL-1β response was only maintained in F2F LPS_1_ male and female groups (offspring from F1 LPS-affected females). Current data may suggest mitochondrial dysfunction from the F2 maternal lineage—mitochondrial stress was showed to activate the NLRP3 complex in aged hematopoietic stem cells, inducing IL-1β production (32). This interpretation is also in line with findings where maternal LPS exposure was associated with mitochondrial DNA abnormalities and metabolic dysregulation in offspring (33). Furthermore, current data specifically suggests that this mechanism may have sex dependency, pointing toward a female-linked intergenerational dysregulation of IL-1β signaling resulting in hyperresponsiveness in subsequent generations.

The previously reported hyperresponsiveness of TNF-α and IL-6 in both F1 and F2 (16), did not show sex specificity, in line with related literature (15, 26, 30, 34, 35). Of further interest, F2 LPS_2_ offspring to two affected parents did not show this hyperresponse. However, in our opinion, it is unlikely that the hyperresponsive maladaptation was abolished; rather, we believe that these data suggests a relatively blunted or delayed reactivity to immune challenge, which may again relate to altered HPA-axis functionality.

### Distinct HPA Status and Functionality in F1 vs. F2 Generation

In terms of sex differences, our current results are in line with literature showing that female mice produce more glucocorticoids than males under basal conditions (36, 37). In the F1 generation, *in utero* LPS exposure elevated CORT in plasma, although this finding was only significant in F1 M LPS and also upregulated GR expression in most of the cell subsets analyzed. The basally elevated CORT and leukocyte GR expression in F1 animals, in combination with slightly elevated cytokine responsiveness, is in line with an interpretation of long-lasting inflammatory phenotype resulting from *in utero* exposure to maternal inflammation, which at this point did not seem to have sex specificity in terms of offspring affected. In further support of our interpretation, intraperitoneal administration of LPS was previously reported not to result in any change in blood CORT levels in F1 offspring (12), suggesting that the upregulated CORT in F1 resulted from maternal inflammation-associated reprogramming.

In F2, male offspring from LPS-affected males (F2M male LPS) showed no generational carryover of CORT hypersecretion, but F2F male LPS did not show the same plasticity, as evidenced by their higher CORT levels. Notably, crossing of the LPS-affected male phenotype with an unaffected female was able to amend the CORT maladaptation, while the LPS-affected female crossed with an unaffected male could not. This implies a maternal-linked inheritance of maladaptation, which further exacerbated the relatively greater GC sensitivity already known to exist in males (5, 38).

The F2 LPS_2_ generation displays an entirely different phenotype with dual parental exposure, when compared to F2 LPS_1_. Specifically of interest is the selective reduction in GR expression in NKT lymphocytes and monocytes in both sexes. Previous studies demonstrated an increase in CD28_null_ NKT lymphocytes in chronic obstructive pulmonary disease, with reduced GR expression. The lack of CD28 co-stimulatory molecule on lymphocytes is linked to lymphocyte senescence and associated with a further increase in their pro-inflammatory/cytotoxic (TNF-α, IFN-γ, granzyme, and perforin production) phenotype and glucocorticoid resistance (39, 40). In systemic lupus erythematosus, similar results are observed in monocytes, with loss of GR on monocytes being the hallmark of steroid resistance, which is also associated with an increased pro-inflammatory cytokine profile (41). The current result may therefore be indicative of a predisposition to developing inflammatory disorders in later life, although we acknowledge that small sample size in the current study precludes firm conclusions at this point.

### Perspectives and Significance

As mentioned, only F2 exhibits a clear depiction of inheritance, as the profile seen in F1 may also, at least in part, have resulted from *in utero* influences (maternal inflammation). In order to extrapolate from our data on F2 in particular, it is important to be reminded of the sequence of events known to occur in chronic stress. Chronic stress is known to result in changes across a continuum, where CORT and GR increases at first, followed by decreased GR. The resulting continued increase in CORT production finally results in adrenal burnout—the point where inflammatory processes escalate due to insufficient GC feedback control.

The current findings suggest that maternal (F0) gestational chronic (LPS-induced) inflammation resulted in transmission of altered HPA programming and maladapted functional immune response, in a sex-dependent manner, in their succeeding lineages. The male offspring (F1) seems to have inherited a maternal-linked maladapted HPA programming that rendered them CORT hyperresponsive,which along with the general increase in GR levels seen in both sexes, may lead to GC resistance. In addition, the F1 male offspring exhibit a relatively exacerbated pro-inflammatory cytokine response. Thus, in our opinion, at the point of adrenal burnout—which for both sexes seem to be brought forward by MPSI, but more pronounced in the males—the males seem to be relatively more vulnerable to inflammation-associated chronic disease. Our finding of higher body mass in LPS-affected F2 males further supports this interpretation. Although this data is in accordance with other studies linking specifically the male sex to maladapted generational transfer, we do acknowledge that translation of these results into a human model is required for validation. For example, these data cannot account for sex differences in terms of behavioral or neurological adaptations to chronic stress. However, given that females, in general, are known to be more likely to make healthier lifestyle choices than men (42, 43), the current data suggests that, in terms of inflammatory disease, the male population may be more susceptible than females.

Although current data and published literature paint a relatively bleak picture for males, in particular, in terms of clinical health outcome, in our opinion, females are not immune to inheriting maldaptations. Although LPS-affected F1 females did not exhibit a more compromised phenotype than their male counterparts, their offspring displayed relatively more maladapted endocrine and inflammatory (IL-1β) immune function, than the F2 offspring from F1 LPS-affected males.

On a more technical note, baseline sex differences observed in control animals also provide valuable information. For example, in F1, females displayed higher peripheral leukocyte counts than males in all subpopulation assessed. However, in F2, females had consistently lower counts for peripheral monocytes and neutrophils than males (although not significant in this small sample number). This confirms that seasonal differences—which has been reported for humans (22, 23)—may also be present in experimentally housed mice and that this variation is sex dependent. In contrast, in both F1 and F2, control females exhibited higher splenic counts for total leukocytes and lymphocytes than control males, suggesting a sex difference that was not influenced by season. This may indicate that indeed, as utilized in the current study, splenic cells may be a better model than peripheral cells, with which to investigate sex differences in the context of immunity. Given the significance of this information, more studies are warranted to confirm these data statistically, as the relatively low n in the current study does not allow firm conclusions in this regard.

## Limitations

In terms of limitations, we were not able to determine more conclusively the contribution of heritability to changes observed, as changes in the epigenome were not assessed. Breeding in the F3 generation in future studies would also further confirm the plasticity and sex specificity of transgenerational epigenetic inheritance. Despite this, we are satisfied that consistent trends and patterns are emerging, which clearly indicates that in investigations of this nature, both sexes should be considered, as mechanisms for maladaptation may differ substantially between the two sexes.

We acknowledge that a significant limitation of this study is the relatively small sample size. However, as mentioned in the *Introduction* section, the data presented here resulted from the re-analysis of data not originally intended to be distinguished between sexes. However, we are confident that we were significantly conservative in our statistical analysis and approach to interpretation of data. Furthermore, the data is in line with relevant published data.

Furthermore, we did not compare F1 to F2 directly, because of the unavoidable seasonal variation in terms of parameters assessed, following our study design. In order to facilitate such a comparison, staggered entry into the protocol (and using higher n) may be a feasible approach.

In terms of translation to human models, we acknowledge the requirement to further validate these results in longitudinal human studies.

## Conclusion

In conclusion, it is clearly acknowledged that exposure to an adverse metabolic milieu before or during gestation accounts for some of the adverse consequences in the resulting affected offspring. Current data suggest that in response to MPSI, the male offspring exhibits a relatively more pro-inflammatory phenotype than the female offspring, as well as a relative glucocorticoid hyperresponsiveness, which is likely because of maternal transgenerational inheritance. In addition, our data on offspring to two affected parents provides preliminary data suggesting cumulative inheritance of maladaptations, which may result in increased chronic disease risk, e.g., because of the immune senescence our data implies. These findings may have substantial implications for the development of disease and require further, more focused investigations.

## Data Availability Statement

The datasets generated for this study are available on request to the corresponding author.

## Ethics Statement

The animal study was reviewed and approved by the Stellenbosch University Animal Research Ethics Committee (Ref # SU-ACUM14-00004).

## Author Contributions

The study was jointly designed by CS and RA. RA conducted the experimental work and data analyses under the supervision of CS. Data interpretation and manuscript preparation were jointly performed by CS and RA.

### Conflict of Interest

The authors declare that the research was conducted in the absence of any commercial or financial relationships that could be construed as a potential conflict of interest.
