# Supplementary material for: In utero Exposure to Maternal Chronic Inflammation Transfers a Pro-Inflammatory Profile to Generation F2 via Sex-Specific Mechanisms
Source: Front Immunol. 2020 Feb 13;11:48. doi: 10.3389/fimmu.2020.00048 (PMC7031653; doi:10.3389/fimmu.2020.00048)
Supplement: Supplementary file 1 [file Data_Sheet_1.docx]

Supplementary Material 1: Gating Strategy

### Leukocyte Glucocorticoid receptor analysis


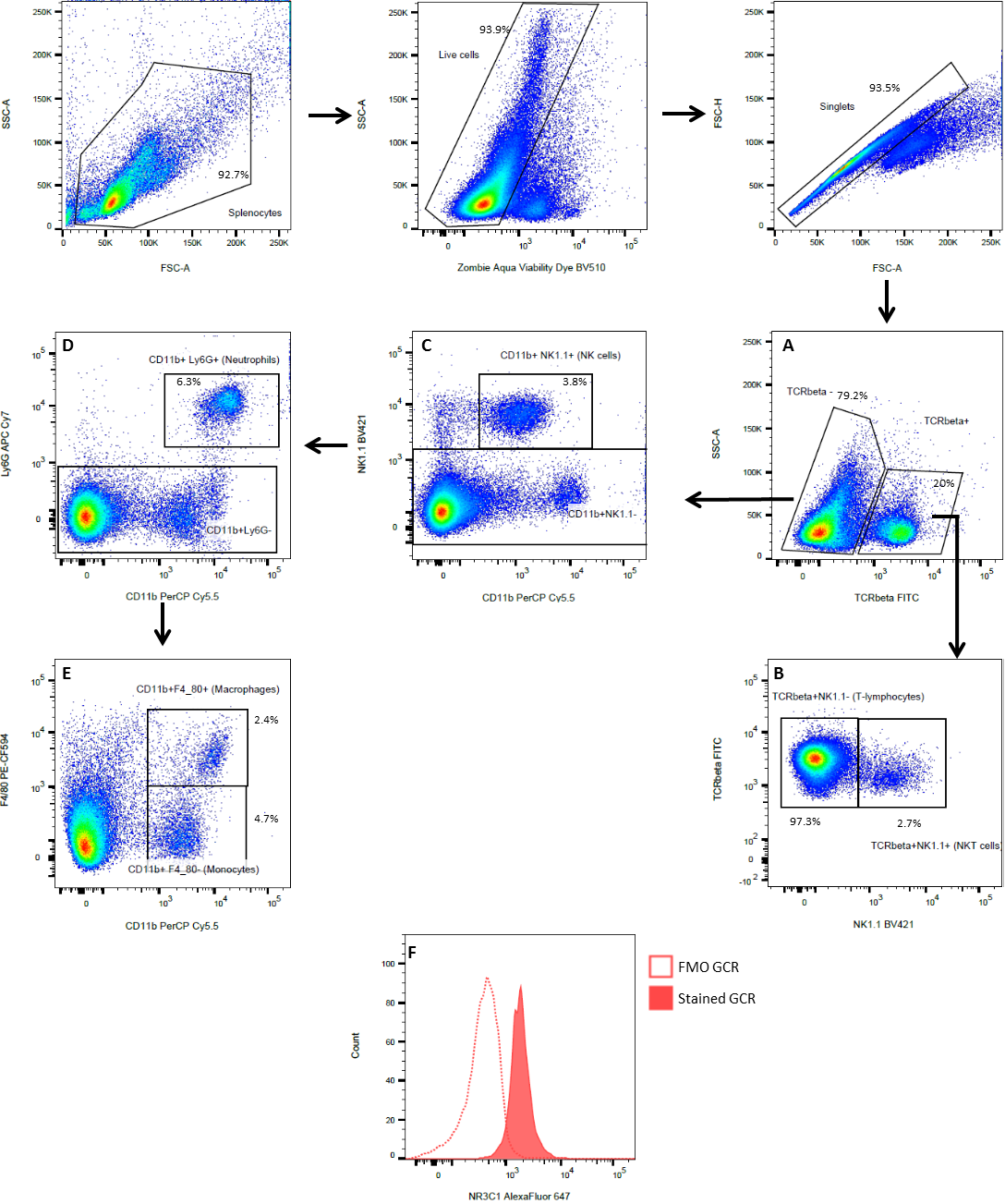


Representative Gating Strategy for Basal Glucocorticoid Receptor analysis. Splenocytes were gated and live cells were gated out using a fixable viability dye. Thereafter, doublets were removed before gating for specific cell subsets. TCRβ was used to differentiate between the lymphoid and non–lymphoid cells of interest (A) and TCRβ+ cells further used to identify T-lymphocytes and NKT lymphocytes (B). Within the TCRβ- population, NK cells (C), neutrophils (D) and monocytes and macrophages (E) were identified, as they all express CD11b as well as their specific markers. Lastly, within each cell population, we looked at the GCR expression (F) as MFI.
